# CaMKII Signaling Stimulates Mef2c Activity *In Vitro* but Only Minimally Affects Murine Long Bone Development *in vivo*

**DOI:** 10.3389/fcell.2017.00020

**Published:** 2017-03-16

**Authors:** Chandra S. Amara, Christine Fabritius, Astrid Houben, Lena I. Wolff, Christine Hartmann

**Affiliations:** Department Bone and Skeletal Research, Medical Faculty of the University of Münster (WWU), Institute of Experimental Musculoskeletal MedicineMünster, Germany

**Keywords:** CaMKII, peptide inhibitor, chondrocyte maturation, hypertrophy, Mef2c, mouse model

## Abstract

The long bones of vertebrate limbs form by endochondral ossification, whereby mesenchymal cells differentiate into chondrogenic progenitors, which then differentiate into chondrocytes. Chondrocytes undergo further differentiation from proliferating to prehypertrophic, and finally to hypertrophic chondrocytes. Several signaling pathways and transcription factors regulate this process. Previously, we and others have shown in chicken that overexpression of an activated form of Calcium/calmodulin-dependent kinase II (CaMKII) results in ectopic chondrocyte maturation. Here, we show that this is not the case in the mouse. Although, *in vitro* Mef2c activity was upregulated by about 55-fold in response to expression of an activated form of CaMKII (DACaMKII), transgenic mice that expressed a dominant-active form of CaMKII under the control of the Col2a1 regulatory elements display only a very transient and mild phenotype. Here, only the onset of chondrocyte hypertrophy at E12.5 is accelerated. It is also this early step in chondrocyte differentiation that is temporarily delayed around E13.5 in transgenic mice expressing the peptide inhibitor CaM-KIIN from rat (rKIIN) under the control of the Col2a1 regulatory elements. Yet, ultimately DACaMKII, as well as rKIIN transgenic mice are born with completely normal skeletal elements with regard to their length and growth plate organization. Hence, our *in vivo* analysis suggests that CaMKII signaling plays a minor role in chondrocyte maturation in mice.

## Introduction

Endochondral ossification is the process underlying the formation of the long bones in the vertebrate limbs (Erlebacher et al., 1995; Kronenberg, 2003). It starts with the condensation of mesenchymal cells that undergo chondrogenic differentiation, forming a cartilage template consisting of immature chondrocytes. These produce an extracellular matrix composed of proteoglycans, glycosaminoglycans, and glycoproteins. This template, which prefigures the future skeletal element, enlarges through chondrocyte proliferation (Akiyama and Lefebvre, 2011). In the next phase, chondrocytes in the center of the cartilage anlage stop proliferating and differentiate into prehypertrophic, *Indian hedgehog* (*Ihh*) expressing chondrocytes. Prehypertrophic chondrocytes enlarge further, becoming hypertrophic chondrocytes, which initiate synthesis of extracellular matrix containing type X collagen, encoded by the *Col10a1* and *Col10a2* genes (Kronenberg, 2003). The transition of chondrocytes from proliferating to prehypertrophic and then to hypertrophic cells is a critical step in determining the growth rate and size of skeletal elements (Kronenberg, 2003).

Previous studies in chicken and mouse identified a complex network of signaling pathways and transcription factors that regulate the different steps during endochondral bone formation (reviewed in Karsenty, 2008; Hartmann, 2009; Lefebvre and Bhattaram, 2010). A central regulatory node in the chondrocyte differentiation program is the Ihh-PTHrP (parathyroid hormone-related peptide) feedback loop (Vortkamp et al., 1996). Ihh, which is part of this feedback loop, is considered a master regulator of chondrocyte maturation and has multiple functions (Kronenberg, 2003; Mak et al., 2008). Numerous transcription factors such as Runx2 and Runx3, Mef2c, and Mef2d, as well as transcriptional co-factors such as β-catenin, promote chondrocyte hypertrophy (Inada et al., 1999; Kim et al., 1999; Hartmann and Tabin, 2000; Yoshida et al., 2004; Arnold et al., 2007; Guo et al., 2009).

It is not yet well established how the activity of these transcription factors is regulated by signaling events. Ca^2+^/calmodulin-dependent kinase II (CaMKII), is a calmodulin (CaM) binding serine/threonine kinase and important for Ca^2+^-mediated signal transduction (Colbran et al., 1989). Most vertebrates possess four different CaMKII genes (α, β, γ, and δ) giving rise to at least 38 different splice variants (Tombes et al., 2003). Two hallmarks distinguish CaMKII from other kinases: firstly, it acts as a multimeric holoenzyme composed of 4–14 heteromeric or homomeric subunits of the different isoforms of the four genes and secondly, its ability to autophosphorylate on the threonine 286 residue upon Ca^2+^/CaM binding (Soderling, 1996; Hudmon and Schulman, 2002; Colbran, 2004; Lantsman and Tombes, 2005; Rosenberg et al., 2006). Autophosphorylation relieves the enzyme from its Ca^2+^/CaM dependence. Alternatively, CaMKII can be activated by methionine oxidation (Erickson et al., 2008).

Various studies suggest that CaMKII signaling may play a role in skeletogenesis. All four genes are expressed in chicken and mouse chondrocytes (Taschner et al., 2008; Li and Dudley, 2009). Studies on human articular chondrocytes have suggested that CaMKII is involved in N-methyl-D-Aspartic acid (NMDA) receptor signaling, which is important for maintaining matrix integrity of joints (Salter et al., 2004; Shimazaki et al., 2006). CaMKII signaling has also been implicated in osteoblast and osteoclast differentiation (Quinn et al., 2000; Zayzafoon et al., 2005). In the chicken, we demonstrated previously using a retroviral system that the expression of a dominant active form of CaMKII (DaCaMKII), which mimics the autophosphorylated form, caused premature ectopic chondrocyte maturation, while the inhibition of CaMKII activity by a peptide inhibitor (rKIIN) delayed the hypertrophic program (Taschner et al., 2008). Li and colleagues suggested that the increasing CaMKII activity in the chondrocytes during their transition from the proliferative to the prehypertrophic state regulates Runx2 and β-catenin activity and thereby promotes chondrocyte hypertrophy (Li et al., 2011).

Retroviral driven expression in the chick system has the disadvantage that all mitotically active cells get infected. So besides the chondrocytes also the soft-tissue is infected. This makes it difficult to distinguish between cell-autonomous and non-cell-autonomous effects. Using a transgene approach in the mouse allowed us to overcome this problem. Hence, in order to gain more specific insights into the potential role of CaMKII in endochondral bone formation, we expressed an activated form of CaMKII (DaCaMKII) or the peptide inhibitor rKIIN under the control of the *Col2a1* promoter primarily in chondrocytes. Based on our observations in the transgenic mouse models we conclude that modulation of CaMKII activity in the mouse has only an effect early in development at the onset of chondrocyte hypertrophy.

## Materials and methods

### Generation of *Col2a1*-transgenic mice

**Figure 1:**
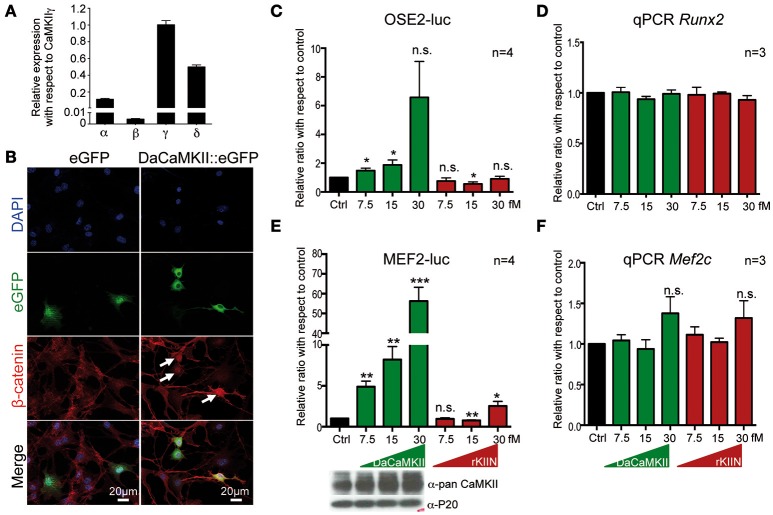
**CaMKII activity translocates β-catenin into the nucleus and regulates Runx2 and Mef2c activity in a dose dependent manner. (A)** qPCR showing the expression levels of the four Camk2 isoforms, α, β, γ, δ, in chondrocytes isolated from E12.5 skeletal elements. Expression levels were normalized to *Gapdh* and *Actb* and ploted relative to the expression of CaMKIIγ. *n* = 3. Error bars indicate ±SEM. **(B)** Epiphyseal chondrocytes from P6 wild-type limbs were transfected with an expression vector encoding DaCaMKII::eGFP or eGFP (control). Nuclear localization of β-catenin (white arrows) was observed in DaCaMKII transfected cells. **(C)** Primary chondrocytes from E13.5 appendicular skeletal elements were co-transfected with 6X Ose2 luciferase (OSE2-luc) reporter and TK-renilla reporter plus increasing amounts of the expression vector encoding DaCaMKII or rKIIN. Ratios of luciferase activity with respect to control (Ctrl, black bar) are plotted as bar charts: DaCaMKII (green bars) and rKIIN (red bars). **(D)** Relative *Runx2* expression levels, determined by qPCR, in primary chondrocytes from E13.5 appendicular skeletal elements co-transfected with increasing amounts of an expression vector encoding DaCaMKII or rKIIN used for the OSE2-Luc assay. **(E)** Primary chondrocytes from E13.5 wild-type appendicular skeletal elements were co-transfected with 3X Mef2 luciferase reporter (MEF2-Luc), TK-renilla reporter, and increasing amounts of the expression vector encoding DaCaMKII or rKIIN. Bar graph showing the ratio of luciferase activity with respect to control (Ctrl, black bar), DaCaMKII (green bars), and rKIIN (red bars) from four independent transfection experiments. The immunoblot below shows the corresponding increase in CaMKII levels. **(F)** Relative *Mef2c* expression levels, determined by qPCR, in primary chondrocytes from MEF2-luc assays co-transfected with increasing amounts of expression vector encoding DaCaMKII or rKIIN. ^*^p < 0.05, ^**^p < 0.01, ^***^p < 0.001, n.s., not significant. Error bars indicate ± SEM.

For the dominant active CaMKII transgene a cassette containing CaMKII-T286D C-terminal fused to eGFP (Taschner et al., 2008) followed at the 3′ end by two SV40polyA sequences was inserted via blunt-end cloning into the BamHI site of a plasmid carrying the *Col2a1* promoter–rabbit β globin intron–*Col2a1* enhancer element (a gift from Yoshi Yamada; (Nakata et al., 1993)). For the rKIIN transgene, a peptide inhibitor for CaMKII from rat (Chang et al., 1998, 2001) fused with eGFP at its N-terminus (Taschner et al., 2008) was cloned accordingly. The final transgenic constructs (Figures 1A,B) were excised with the restriction enzymes AfllII and SwaI, purified on agarose gel and eluted with DNA microinjection buffer. The linear transgene cassettes were microinjected into the pronucleus of B6CBAF_1_ zygotes in the transgene facility of the IMP, Vienna, Austria (Hogan et al., 1994). The zygotes were implanted into pseudo-pregnant mice to obtain transgenic founder lines. To maintain the transgenic lines in a pure genetic background, the founder lines were crossed with C57Bl/6J females and the subsequent generations were backcrossed with C57Bl/6J animals over at least eight generations. Genotyping of transgenic mice and embryos was performed by PCR using ear biopsies and material from the embryonic tail respectively, in combination with transgene-specific primer pairs (listed in Supplementary Table 1). Transgene-copy numbers were determined using genomic DNA from ear biopsies of the different transgenic lines (two independent samples per line) by qPCR and normalized to actin and control genomic DNA from mice carrying one copy of the transgene in the Rosa26 locus (Amara and Hartmann, unpublished; Ballester et al., 2004). Primers are listed in Supplementary Table 2. Mouse experiments were performed in accordance with local, institutional and national regulations under the following licenses (84-02.05.2012.260, 84-02.04.2015.A278, 84-02.05.50.15.022).

### Mouse husbandry and embryo processing

For timed pregnancies, heterozygous transgenic mice were interbred overnight and the plug day was designated as embryonic day 0.5 (E0.5). Embryos were harvested at the required stages, dissected and fixed overnight in 4% paraformaldehyde (PFA). For stages E18.5 and older, the skin above the limbs was removed prior to fixation. Fixed limbs were removed, washed in PBS, and tissue was dehydrated using increasing ethanol concentrations (25, 50, 75, 100%).

### *In situ* hybridization, histology and skeletal preparations

For *in situ* hybridization (ISH) and histological staining on sections, limbs were processed into paraffin using the Excelsior ES Vacuum Infiltration Processor (Thermo Scientific), embedded in paraffin and sectioned at 5 μm. Non-radioactive ISH were performed using digoxigenin (DIG)-labeled anti-sense RNA probes as previously described (Murtaugh et al., 2001). Probes for type 2 collagen (*Col2a1*), indian hedgehog (*Ihh*), osteocalcin (*Ocn*), osteopontin (*Opn*), type 1 collagen (*Col1a1*), and type 10 collagen (*Col10a1*) have been published previously (Hill et al., 2005). The *Gfp* probe was generated using a plasmid containing the eGFP coding region. All probes are available upon request. Histological stainings such as alcian blue, alcian blue/von Kossa, and hematoxylin/eosin were performed as previously described (Houben et al., 2016). Skeletal preparations were performed on 6 day old pups which were sacrificed by decapitation, skinned, eviscerated, fixed in 95% EtOH, and double-stained for alcian blue and alizarin red (McLeod, 1980). The length of isolated humeri and femora was calculated by using Zeiss image analysis software.

### RNA isolation and qPCR

The stylo- and zeugopod regions of forelimbs were isolated from embryos at the required stage. Skin and soft tissue was removed and skeletal elements were dispersed using the Polytron PT 1200 E manual disperser with the aggregate PT-DA 03/2 EC-E50 (Kinematica). The RNA was isolated using the QIAGEN RNeasy micro kit according to the manufacturer's instructions. A total of 500 ng RNA was reverse transcribed using PrimeScript RT reagent kit (Takara, #RR037A) with oligo dT primer. For qPCR cDNA was diluted 1:10 in water. 3 μl of diluted cDNA were mixed with 1 μl of primer mix and 10 μl SYBR Premix Ex TaqII (Tli RNaseH plus) (Takara, #RR820Q) in a final volume of 20 μl. Gene expression was monitored using the BioRad CFX96 cycler and the following protocol: 95°C 10 min, 45 × (95°C 30 s, 60°C 30 s, 72°C 45 s + plate read), 72°C 5 min, melting curve: 55°C–99°C in 0.5°C increments for 5 s + plate read. qPCR analysis was performed in duplicates and results were produced from at least three independent samples. Expression values were calculated using the comparative ΔC(t) method and normalized to *Gapdh* and *Actb* expression. For primer sequences and product sizes see Supplementary Table 3.

### Isolation and cultivation of primary chondrocytes

E13.5 appendicular skeletal elements from the stylo- and zeugopod were dissected in PBS supplemented with 1% P/S followed by a digestion with 0.1% type II collagenase (Gibco, #17101-015) and 0.3% dispase (Gibco, #17105-041) in medium (DMEM/F-12; 1% P/S) for a period of 60 min shaking at 100 rpm in a CO_2_ incubator. Cells were filtered through a cell strainer, centrifuged and washed with culture medium (DMEM/F-12, 1% P/S, and 10% FCS). Cell pellets were then resuspended in culture medium, plated at 2.5–3 × 10^4^ cells/cm^2^ in tissue culture flasks and grown for 5 days.

### Luciferase assays

Primary chondrocytes were seeded at a density of 3 × 10^4^ cells/well in 48 well plates 1 day prior to transfection. Cells were transfected with the pGL4.23-Mef2-luc (a gift from Eric Olson) or the pGL4.10-Ose2-luc reporter plasmid (a gift from G. Karsenty) in combination with pRL-TK (Promega) as a control and pCDNA3.1-DaCaMKII or pCDNA3.1-rKIIN expression vectors. Total amounts of transfected plasmids (equivalent to 52 fM) in each group were adjusted by adding empty vector (pCDNA3.1+). Transfection was performed using jetPRIME™ (Polyplus transfection) according to manufacturer's instructions. Luciferase activities were measured 48 h after transfection following the protocol by (Hampf and Gossen, 2006). Luciferase measurements were normalized to the corresponding renilla activities for transfection efficiency. Experiments were performed in triplicates and repeated at least three times.

### Immunofluorescence

For immunofluorescent staining on cells, primary chondrocytes isolated from P6 C57Bl/6J knee epiphyseal regions were seeded on 0.1% gelatin coated glass coverslips. Cells were transfected with DaCaMKII::eGFP and eGFP expression vectors (equivalent to 0.12 pmol) using jetPRIME™ (Polyplus transfection). Forty-Eight hours after transfection, cells were fixed with 4% PFA/PBS for 20 min, treated with 0.5% TritonX-100 for 5 min and blocked with 10% normal goat serum (NGS) in PBS for 1 h at room temperature (RT). Incubation with β-catenin antibody (1:200 in 2% NGS/PBS, BD Biosciences, #610154, RRID:AB_397555) was performed overnight at 4°C. Coverslips were subsequently incubated with Alexa Fluor 647 goat anti-mouse IgG (Molecular Probes) at 1:200 in 2% NGS/PBS. Nuclei were counterstained with DAPI.

### Immunoprecipitation by magnetic beads

HEK293 cells were co-transfected with Mef2c-Myc and DaCaMKII::eGFP at 4:3 ratio or FLAG-HDAC4 and DaCaMKII::eGFP at 1:3 ratio using CaPO_4_ transfection method. Cells were lysed in ice-cold lysis buffer (50 mM Tris-HCl pH 7.5, 150 mM NaCl, 1% NP-40 with proteinase and phosphatase inhibitors). After centrifugation, a total amount of 150 μg of protein from the supernatant was subjected to immunoprecipitation using 25 μl magnetic beads coupled with GFP monoclonal antibody (MBL, #D153-11) under the following conditions: 15 min at 4°C on a rotating wheel (10 rpm), washed 4 times with wash buffer (50 mM Tris-HCl pH 7.5, 150 mM NaCl, 1% NP-40) for 5 min at RT. For the pull-down of FLAG-HDAC4 using GFP-magnetic beads the conditions were as following: 500 μg of protein from the supernatant was subjected to immunoprecipitation using 50 μl magnetic beads and bound for 45 min at 4°C on a rotating wheel (10 rpm), followed by 3 washes (50 mM Tris-HCl pH 7.5, 150 mM NaCl, 0.05% NP-40) for 30 s at RT. Magnetic beads were then boiled for 2 min in 25 μl Laemmli's sample buffer and supernatant of the beads was loaded on a 10% SDS-PAGE to separate the immunoprecipitated proteins.

### Immunoblots

Transfected primary chondrocytes were lysed with ice-cold lysis buffer (50 mM Tris-HCl pH 7.5, 150 mM NaCl, 1% NP-40 with proteinase and phosphatase inhibitors). 50 μg of protein lysates were run on a 10% SDS-PAGE and transferred to a 0.45 μm PVDF-membrane by semi-dry transfer (PerfectBlue™, PeqLab). The membrane was blocked with 5% milk and incubated with the appropriate primary antibodies anti-GFP (1:15000, Abcam, #ab13970, RRID:AB_300798), anti-Myc (1:1000, Cell Signaling Technology, mAb #2276, RRID:AB_331783), pan-CaMKII (1:1000, Cell Signaling Technology, mAb #4436S, RRID:AB_10545451), anti-Flag (1:1000, Sigma-Aldrich, #F1804, RRID:AB_262044), and proteasome 20 (P20) (1:15000, Abcam, #ab3325, RRID:AB_303706), followed by incubation with the respective, species-specific HRP-coupled secondary antibodies (1:1000 and 1:5000). ECL substrate was used for signal development (Amersham, #RPN2106) on X-ray film (Amersham).

### Image acquisition

Histological images were acquired using Zeiss AxioImager M2 equipped with an AxioCam MRc 6.45 μm color camera (Zeiss, Jena). Images of embryos were acquired using a Zeiss Stereo discovery V8 equipped with Zeiss plan Apo S, 0.63X lens. Immunofluorescent images were acquired using Zeiss AxioImager M2 equipped with an Apotome2 and an AxioCam MRm 6.45 μm monochromatic camera (Zeiss, Jena) using the Zen software (Zeiss, Jena).

### Statistical analysis

All statistical analyses were performed using GraphPad Prism software 6.0 (RRID: SCR_002798). Data are displayed as mean values ± standard error of the mean (SEM). Statistical significance of differences (*P*-value) was determined by the two-tailed, unpaired Student's *t*-test.

## Results

### *In vitro*: CaMKII signaling affects β-catenin localization and alters Runx2 and Mef2c activity

Based on previous studies in chicken, potential molecular mechanisms of how CaMKII signaling regulates chondrocyte hypertrophy have been suggested (Li et al., 2011). These included increased nuclear localization of β-catenin and Runx2 in chondrocytes with activated CaMKII signaling (Li et al., 2011). Thus, we investigated whether the molecular mechanism in mouse chondrocytes may be similar. Mouse chondrocytes, like chicken chondrocytes, express all four CaMKII isoforms, and also here the CaMKIIβ isoform was expressed at lower levels than the other three isoforms (Figure 1A, see also Taschner et al., 2008; Li et al., 2011). Similar to what was observed in chicken, β-catenin was localized in the nucleus in mouse chondrocytes transfected with a DaCaMKII::GFP expression plasmid (Figure 1B, white arrows). This was not observed in the GFP-transfected control cultures (Figure 1B, left panel). We also analyzed whether Runx2 transcriptional activity was altered by the presence of DaCaMKII or rKIIN in mouse chondrocytes. For this, the Runx2-dependent OSE2-luc reporter (Ducy and Karsenty, 1995) was co-transfected into primary chondrocytes with increasing amounts of either DaCaMKII or rKIIN expression plasmid. OSE2-luc reporter activity was upregulated in response to increasing amounts of DaCaMKII, while expression of rKIIN led to a downregulation of the reporter activity in a concentration independent manner (Figure 1C). To rule out the possiblity that modulation of CaMKII activity affects the expression levels of Runx2 itself, we examined the *Runx2* expression levels in the OSE2-luc assay by qPCR. Here, *Runx2* expression was not affected in cells transfected with the DaCaMKII or rKIIN expression plasmids (Figure 1D).

**Figure 2:**
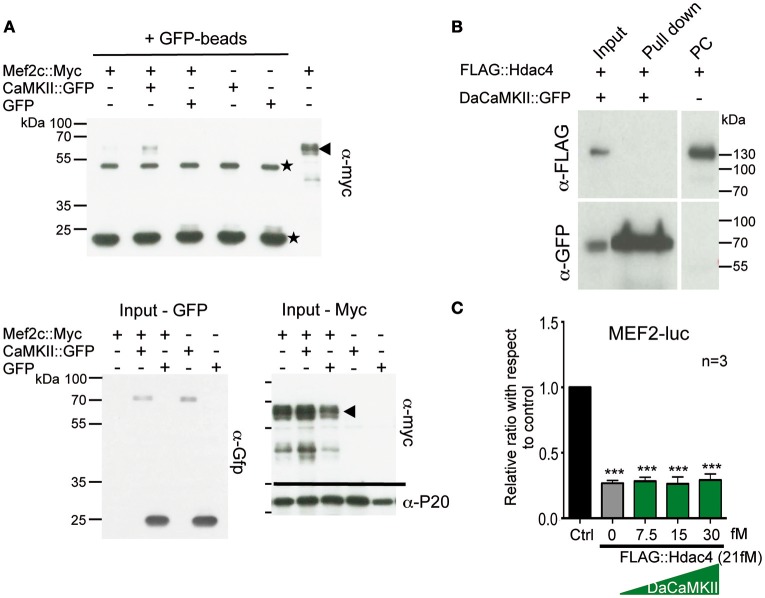
**CaMKII physically interacts with Mef2c but not with HDAC4 and does not modulate Hdac4 activity. (A)** Representative immunoblot of a co-immunoprecipitation assay using GFP-magnetic beads and lysates from HEK293 cells co-transfected with Mef2c::Myc and CaMKII::GFP expression plasmids or Mef2c::Myc and GFP expression plasmids analyzed with anti-myc tag antibody (*n* = 3). An interaction was observed in the Co-IP of Mef2c::Myc with CaMKII::GFP (lane 2; indicated by the arrow at the hight of the Mef2c::Myc signal). The two unspecific signals due to the IgG heavy and light chains, are indicated by the stars. Below the input for the Gfp- and Myc-tagged proteins used in the co-IP and the loading control with anti-P20 are shown. **(B)** Representative immunoblot of a co-immunoprecipitation assay using GFP-magnetic beads on lysates from HEK293 cells co-transfected with FLAG::Hdac4 and CaMKII::GFP expression plasmids analyzed with anti-myc tag antibody. No interaction was observed between DaCaMKII::eGFP and Hdac4 (lane 2), *n* = 2. **(C)** Primary chondrocytes from E13.5 wild-type appendicular skeletal elements were co-transfected with MEF2-luc, TK-renilla reporter and expression vectors encoding FLAG-Hdac4, and DaCaMKII (increasing amounts). The ratio of luciferase activity with respect to control (Ctrl) is plotted in the bar graph: control (black bar), FLAG::Hdac4 (gray bar) and FLAG::Hdac4 together with DaCaMKII (green bars), *n* = 3. *n* refers to the number of independent biological samples. ^***^p < 0.001. Error bars indicate ± SEM.

Another transcription factor promoting hypertrophy is Mef2c (Arnold et al., 2007). The MEF2-luc reporter (Naya et al., 1999) was activated over 50-fold by DaCaMKII at the highest concentration used (Figure 1E). An increase in CaMKII protein levels corresponding to the increased amount of transfected expression plasmid was demonstrated by western blot (Figure 1E, lower panel). The DaCaMKII expression levels were also determined by qPCR using a primer that detects the mouse as well as the transgenic rat Camk2a transcript. Here, the Camk2a expression levels were normalized to the endogenous Camk2a expression levels in control-transfected chondrocytes. This quantitative approach revealed an over 100-fold increase in Camk2a levels the cells transfected with 30 fM DaCaMKII (Supplementary Figure 1). Overexpression of rKIIN on the other hand, led, with the exception of the highest concentration, to a downregulation of the MEF2-luc reporter activity independent of the concentration (Figure 1E). To our surprise, at the highest concentration even a two-fold activation was observed (Figure 1E). Again, the endogenous *Mef2c* expression levels were not significantly affected by either DaCaMKII or rKIIN expression in the transfected primary chondrocytes (Figure 1F). Given the strong effect on Mef2c activity we tested whether CaMKII may directly interact with Mef2c. Co-immunopre-cipitation assays in HEK293 cells using tagged proteins revealed that the activated form of CaMKII physically interacts with Mef2c (Figure 2A). Here we used GFP-magnetic beads and pulled on the DaCaMKII::GFP fusion protein and detected bound Mef2c::Myc (Figure 2A). Conversely, using Myc-magnetic beads and pulling on Mef2c::Myc the DaCaMKII::GFP fusion protein was detected by immunoblot in the bound fraction (data not shown).

Given previous findings that (a) the CaMKIIδ isoform can interact with and influence histone deacetylase 4 (Hdac4) activity in cardiomyocyte hypertrophy (Backs et al., 2006, 2009), (b) Hdac4 controls chondrocyte hypertrophy by interacting with and inhibiting the activity of Runx2 and Mef2c (Vega et al., 2004; Arnold et al., 2007), and (c) our observation that DaCaMKII modulates Mef2c as well as Runx2 activity, we addressed whether DaCaMKII also physically interacts with Hdac4 or whether it alters its repressive activity. For this, HEK293 cells were co-transfected with FLAG::Hdac4 and DaCaMKII::GFP. Co-IP studies with GFP-coupled magnetic beads revealed no physical interaction for Hdac4 in the pull-down lysate (Figure 2B). Luciferase assays on primary chondrocytes transfected with MEF2-luc, Mef2c and Hdac4 resulted in the inhibition of Mef2 activity. Yet, this inhibitory effect was not altered by the addition of increasing amounts of DaCaMKII (Figure 2C). This suggests that the DaCaMKII does not modulate Hdac4 activity. In essence, our results show that the activated form of CaMKII positively influences the activity of the transcription factors Mef2c and to a lesser extent Runx2 activity and that this effect is probably not mediated via an inhibitory effect on Hdac4. Furthermore, CaMKII physically interacts with Mef2c suggesting that it may possibly modulate its transcriptional activity by phosphorylation.

### *In vivo*: generation of chondrocyte specific transgenic mice

**Figure 3:**
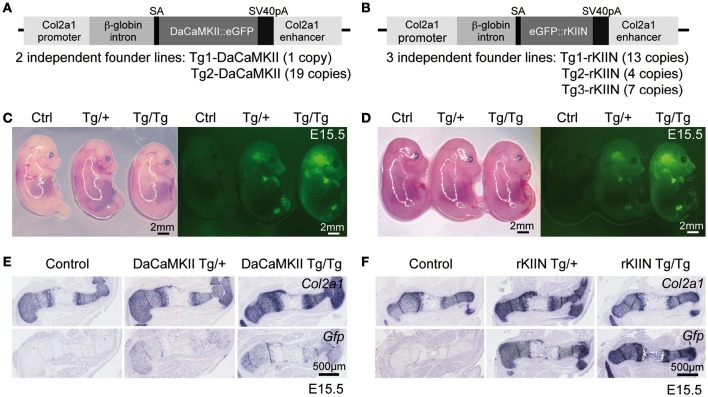
**Overview of transgenic constructs and their chondrocyte-specific expression in transgenic embryos. (A,B)** Schematic view of constructs containing either the DaCaMKII::eGFP **(A)** or eGFP::rKIIN **(B)** downstream of the β-globin intron under the control of the *Col2a1* promoter/enhancer region. The number of independent transgenic lines achieved for each construct and the corresponding copy numbers in the genome are indicated below. **(C,D)** E15.5 littermates from intercrosses of DaCaMKII::eGFP transgenic mice **(C)** and from intercrosses of eGFP::rKIIN transgenic mice **(D)** imaged under white light and fluorescence. The GFP signal is detected in the cartilaginous skeletal elements of limbs, skull, and vertebrae. Note that the GFP signal is more intense in the homozygous (Tg/Tg) embryos compared to heterozygous (Tg/+) littermates, while only weak auto-fluorescence is detected in the control (non-transgenic, +/+) littermates. Images were taken at the same magnification under fluorescent stereomicroscope (Zeiss). **(E,F)** Non-radioactive *Col2a1* and *Gfp* ISH on E15.5 humeri isolated from DaCaMKII-tg mice **(E)** and isolated from rKIIN-tg mice **(F)** and corresponding control (non-transgenic, +/+) littermates showing that the *Gfp* riboprobe signal overlaps almost completely with that of *Col2a1*. Signal intensity for the *Gfp* anti-sense riboprobe differs between heterozygous and homozygous transgenic embryos. SA, splice acceptor site; pA, poly A.

In order to determine the *in vivo* role of CaMKII modulation in mouse endochondral ossification, we employed a transgenic approach using the *Col2a1* promoter/enhancer element to drive transgene expression in chondrocytes (Figures 3A,B). From previous studies in the chicken we knew that eGFP tagged transgenes are functional (Taschner et al., 2008). Hence, in order to visualize transgene expression and to facilitate the distinction between heterozygous and homozygous embryos, eGFP tagged transgenes were used. For the DaCaMKII::eGFP construct two independent transgenic founder lines (Tg1 and Tg2-DaCaMKII) were established and for the eGFP::rKIIN construct three independent founder lines were obtained (Tg1, Tg2, and Tg3-rKIIN; Figures 3A,B). All founder lines transmitted the transgene to the F1 generation. The transgenic lines differed in the number of transgenes integrated in the genome (Figure 3A). For a first characterization of the transgenic lines, heterozygous transgenic mice of the independent founder lines were intercrossed to isolate embryos at embryonic day (E) 15.5. Contrary to our expectations, no obvious gross morphological differences were detected within the offspring of the various intercrosses (Figures 3C,D). Genotyping of the offspring was performed examining the GFP intensity using a fluorescent stereomicroscope and confirmed by conventional PCR-based genotyping (Figures 3C,D; data not shown). Heterozygous and homozygous transgenic embryos were classified based on the fluorescence intensity (Figures 3C,D). As expected, GFP activity was detected in the cartilaginous regions of the skeletal elements, particularly visible in the skull and limbs at sites corresponding to *Col2a1* expression domains (Figures 3C,D; Cheah et al., 1991). This was confirmed by ISH with RNA antisense probes for *Col2a1* and *Gfp* on adjacent sections of transgenic limbs (Figures 3E,F). As expected, no *Gfp* signal was detected in humeri of non-transgenic (wild-type) littermates (Figures 3E,F). These results demonstrated that the transgene is indeed expressed in a *Col2a1*-promoter/enhancer-dependent manner. Furthermore, the GFP signal intensity in embryos as well as the *Gfp* ISH signal on sections allowed us to distinguish heterozygous and homozygous transgenic littermates.

**Figure 4:**
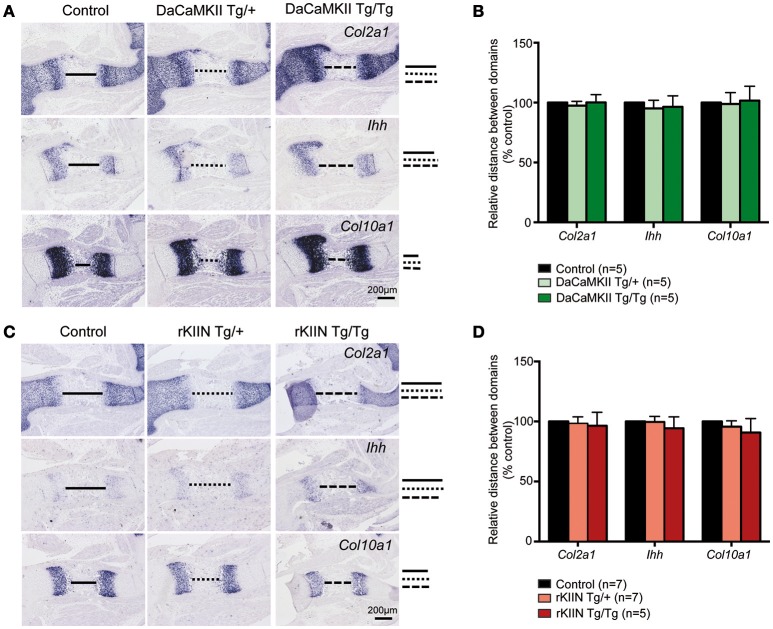
**Modulation of CaMKII activity has no effect on the progression of chondrocyte maturation. (A,C)** Analysis of E15.5 DaCaMKII **(A)** and rKIIN **(C)** transgenic and respective control (non-transgenic) littermate humeri. **(A)** ISH for *Col2a1, Ihh*, and *Col10a1* on sections of E15.5 humeri from control, DaCaMKII hetero- (Tg/+) and homozygous (Tg/Tg) littermates, showing no major alterations in the expression levels or the sizes of the expression domains of these three markers. **(B)** Quantification of the relative distance between the *Col2a1, Ihh*, and *Col10a1* domains revealed no significant differences for these markers. **(C)** ISH for *Col2a1, Ihh*, and *Col10a1* on sections of E15.5 humeri from control, rKIIN hetero- (Tg/+) and homozygous (Tg/Tg) littermates revealed no obvious alterations. **(D)** Quantification of the relative distance between the *Col2a1*, the *Ihh*, and the *Col10a1* domains confirmed the absence of a significant difference. The distance between the domains in control was set to 100%. **(B,D)** The number of independent biological samples is referred to by *n*. The results are not significant. Error bars indicate ± SEM.

In the chicken a subset of the chondrocytes that expressed DaCaMKII prematurely differentiated into prehypertrophic and subsequently into hypertrophic chondrocytes outside their normal expression domains (Taschner et al., 2008; Li et al., 2011). Hence, we performed ISH on E15.5 sections through the limbs using *Col2a1* as a marker for more immature chondrocytes, *Ihh* as a marker for prehypertrophic cells, and *Col10a1* as a marker for hypertrophic cells. Yet, no expression of the maturation markers *Ihh* or *Col10a1* was observed outside their normal expression domains and no obvious differences were observed with respect to the individual domain sizes, distance between the domains, or the expression levels of these three markers comparing non-transgenic with heterozygous and homozygous DaCaMKII-tg littermates (Figures 4A,B). Similar, negative results were obtained performing the analogous analysis on sections of E15.5 humeri from non-transgenic, heterozygous, and homozygous rKIIN-tg littermates (Figures 4C,D). Here, we did not observe an obvious delay in chondrocyte maturation, as one could have expected based on the effect of rKIIN overexpression in chicken.

**Figure 5:**
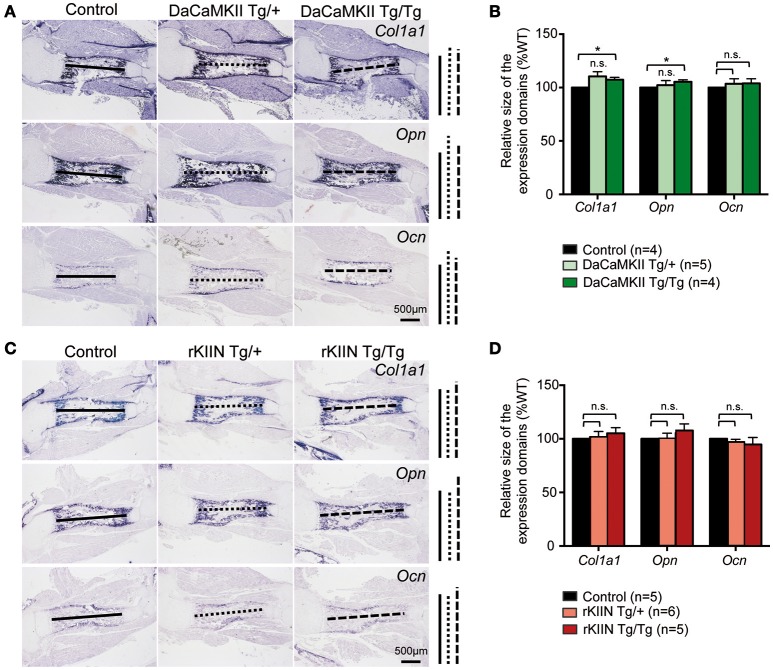
**Modulation of CaMKII activity does not affect the endochondral ossification process. (A,C)** Representative images of ISH for the osteoblastic markers *Col1a1, Opn*, and *Ocn* on sections through E18.5 humeri of DaCaMKII- **(A)** and rKIIN-tg **(C)**, and their respective littermate, non-transgenic controls. **(B,D)** Bar diagram representing relative size (longitudinal expansion) of the *Col1a1, Opn*, and *Ocn* expressing domains (zone of ossification) in the humeri of control (non-transgenic), DaCaMKII- **(B)** and rKIIN-tg **(D)** E18.5 embryos. **(B,D)** The length between the domains in the control humeri was set to 100% to account for developmental differences between litters. *n* refers to the number of independent biological samples. ^*^p < 0.05, n.s., not significant. Error bars indicate ± SEM.

The final step in endochondral ossification is the remodeling of the cartilage template into bone. Thus, in order to see whether this remodeling process was altered, we analyzed the longitudinal extension of the ossified zone by performing ISH analysis for the osteoblastic markers *collagen 1 (Col1a1), osteopontin (Opn)*, and *osteocalcin (Ocn)* on sections of E18.5 DaCaMKII- and rKIIN-tg humeri and corresponding non-transgenic littermates. No significant alteration to the longitudinal extension of the ossified zone for either of the three analyzed markers was observed when comparing the humeri of the transgenic embryos expressing either DaCaMKII (Figures 5A,B) or rKIIN with humeri of their respective, non-transgenic controls (Figures 5C,D). Similar to what we had observed at E15.5, no morphological alterations with regard to the shape, organization or appearance of the prehypertrophic and hypertrophic chondrocytes were detected in the growth plates of the E18.5 DaCaMKII- and rKIIN-tg humeri compared to their corresponding non-transgenic littermate controls (Supplementary Figures 2A,B). In addition, the total length, as well as the length of the mineralized region was measured in alician blue/alizarin red stained humeri of 6-day-old pups. Here again, no differences regarding the two parameters were observed in comparison to non-transgenic littermate controls (Supplementary Figure 3).

### Modulation of CaMKII signaling affects the onset of chondrocyte maturation

**Figure 6:**
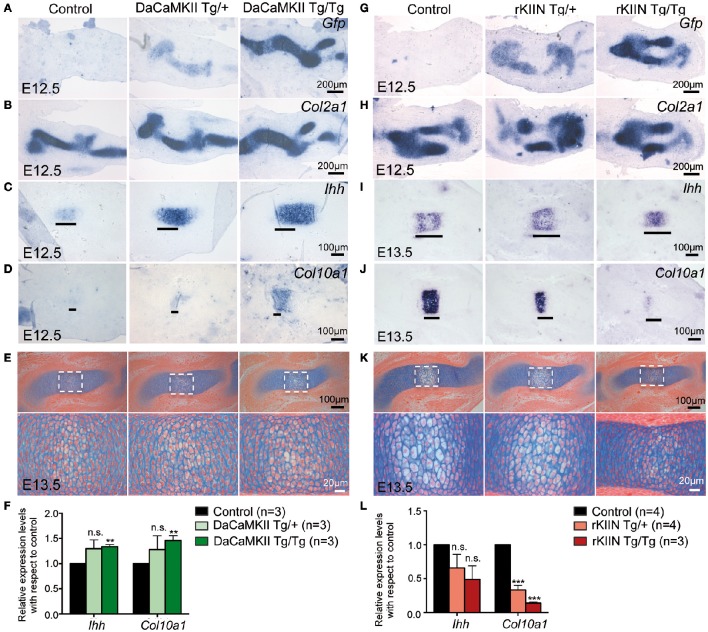
**Modulation of CaMKII signaling affects the onset of chondrocyte maturation in early skeletal development. (A–D)** Representative, alternating sections through humeri of E12.5 control (non-transgenic, +/+), hetero- (Tg/+), and homozygous (Tg/Tg) DaCaMKII-tg littermates. **(A)** *Gfp* ISH revealing transgene expression in heterozygous and homozygous animals but not in non-transgenic littermate controls. **(B)** *Col2a1* ISH confirming that the transgene expression is restricted to the *Col2a1* positive domain. **(C)** *Ihh* ISH defining the prehypertrophic zone, which is enlarged in the homozygous and heterozygous transgenic embryos. **(D)** *Col10a1* ISH marking the onset of hypertrophic chondrocyte differentiation. *Col10a1* expression was more intense in the transgenic embryos compared to control (non-transgenic) littermates. **(E)** Alcian blue/eosin stained representative images of sections through E13.5 humeri of control (non-transgenic; +/+), hetero- (Tg/+), and homozygous (Tg/Tg) DaCaMKII-tg embryos. Images below show magnifications of the boxed areas in the panel above. Due to their increase in volume and vacuolization of the cells, the hypertrophic chondrocytes appear whiter. The region of hypertrophy is enlarged in the transgenic humeri compared to wild-type littermate control. **(F)** qPCR analysis for *Ihh* and *Col10a1* normalized to *Gapdh* and *Actb* using material from E12.5 control, hetero- and homozygous DaCaMKII-tg forelimbs reveals a dose-dependent increase in the expression of these two genes. Gene expression levels are plotted relative to control. **(G–J)** Representative, alternating sections through humeri of E12.5 **(G,H)** and E13.5 **(I,J)** control (non-transgenic; +/+) and hetero- (Tg/+), and homozygous (Tg/Tg) rKIIN-tg littermates. **(G)** *Gfp* ISH revealing transgene expression in heterozygous and homozygous animals but not in non-transgenic littermate controls. **(H)** *Col2a1* ISH confirming that the transgene expression is restricted to the *Col2a1* positive domain. **(I)** *Ihh* ISH defining the prehypertrophic zone, which is reduced in a transgene dose-dependent manner in the hetero- and homozygous transgenic embryos. **(J)** *Col10a1* ISH defining the hypertrophic zone, showing that hypertrophic differentiation is even more delayed. **(K)** Histological alcian blue/eosin stained representative images of sections from control, hetero- and homozygous rKIIN embryos: here, fewer and smaller hypertrophic, whiter cells are visible in hetero- and homozygous transgenic humeri compared to wild-type littermate controls. **(L)** qPCR analysis for *Ihh* and *Col10a1* normalized to *Gapdh* and *Actb* using material from E13.5 control, hetero- and homozygous rKIIN-tg forelimbs revealing a dose-dependent reduction in the expression of *Ihh* and *Col10a1*. Gene expression levels are plotted relative to control. **(F,L)** *n* refers to the number of independent biological samples. ^**^p < 0.01, ^***^p < 0.001, n.s., not significant. Error bars indicate ± SEM.

As there were no obvious effects visible in the older mouse limbs, we addressed whether early steps of skeletogenesis would potentially be affected. In the forelimb the anlagen of humerus, radius and ulna are visible at E11.5. At this stage the cells in the center of the humerus begin to express *Ihh* (data not shown), while they start to express *Col10a1* around E12.5. In humeri of E12.5 DaCaMKII transgenic limbs the marker for prehypertrophic chondrocytes, *Ihh*, was expressed in a slightly broader domain compared to non-transgenic, control littermates (Figures 6A–C). The effect was more prominent in humeri of homozygous (6/8) than heterozygous (8/11). When we analyzed the expression of *Col10a1*, as a marker for hypertrophic chondrocyte differentiation, weak *Col10a1* expression was first detected in the humerus at E12.5-E13.0 in wild-type limbs. In the transgenic specimens, in which we had observed a broadend *Ihh* domain we noticed on adjacent sections a more intense staining for *Col10a1* in a broader domain (Figure 6D). Again the effect was more obvious in the homozygous specimens. Next we asked whether the increased *Col10a1* expression was accompanied by the histological appearance of hypertrophic chondrocytes. For this, we examined E12.5 and E13.5 limbs by alcian blue staining. On alcian blue stained sections, the hypertrophic chondrocytes within the cartilage elements appear lighter in color due to their increase in size and vacuolization. Histological examination of E12.5 humeri (*n* = 2) revealed no apparent signs of hypertrophic chondrocyte differentiation in the transgenic limbs (Supplementary Figure S4A). In E13.5 DaCaMKII-tg humeri, a moderate size increase of the zone of hypertrophic chondrocytes was detected compared to the non-transgenic control (Figure 6E, *n* = 3). Consistent with the *in vivo* ISH results, qPCR analysis of material from E12.5 DaCaMKII transgenic and non-transgenic control limbs revealed an increase in *Ihh* and *Col10a1* expression levels in the transgenic limbs in comparison to the control (Figure 6F). These results suggest that the onset of chondrocyte maturation is slightly accelerated in the transgenic limbs.

We then analyzed rKIIN transgenic limbs at equivalent stages. At E12.5, no obvious differences were detected with respect to the size or intensity of the *Ihh* expression domains between transgenic and non-transgenic embryos (Supplementary Figure S4B). However, at E13.5 the two expression domains of *Ihh* were not yet separated in the humeri of homozygous transgenic animals and still closer together in the heterozygous transgenic animals compared to non-transgenic littermate controls (Figure 6I). Furthermore, the *Col10a1* expression domain was reduced in size in the humeri of the transgenic animals compared to non-transgenic littermate controls (Figure 6J). Together, his suggests that the onset of chondrocyte maturation is slightly delayed when CaMKII signaling is antagonized. Again, the effect was more pronounced in homozygous (9/9) than in heterozygous transgenic specimens (11/13) compared to non-transgenic specimens (Figures 6I,J). Histological examination of alcian blue stained E13.5 humeri confirmed that the decrease in the size of the *Col10a1* expression domain in homo- and heterozygous humeri was associated with reduced hypertrophic chondrocyte differentiation (Figure 6K). Quantification of *Ihh* and *Col10a1* expression levels by qPCR using material from E13.5 forelimbs corroborated the dose-dependent effects of CaMKII-signaling inhibition, which was statistically significant with respect to *Col10a1* expression (Figure 6L). Together, our results suggest that modulation of CaMKII signaling in the mouse affects the onset of chondrocyte maturation during the early stages of endochondral ossification.

ISH with a *Gfp* riboprobe confirmed transgene expression at all stages examined and at least according to the ISH transgene expression was not silenced after E13.5 (Supplementary Figure 5). In order to determine why the expression of the DaCaMKII and rKIIN-transgenes may not lead to a major phenotype at later stages we examined the transgene expression level in the two DaCaMKII-transgenic lines, DaCaMKII-Tg1 (1 copy) and DaCaMKII -Tg2 (19 copies), using qPCR and RNA isolated from the chondrogenic elements of E12.5 limbs. Transgene expression levels were estimated by comparing the DaCaMKII::GFP transgene expression level to the endogenous expression levels of the four CaMKII isoforms and the levels were normalized to the *Camk2g* expression level. Here, we noticed that the transgene expression levels in the Tg1 line were similar to the *Camk2a* endogenous levels in the wild-type control. Yet, they were lower than the endogenous expression levels of the γ and δ isoforms. Interestingly, the *Camk2a* expression levels were increased significantly in the transgenic skeletal elements of the DaCaMKII-Tg1 line. In contrast, transgene expression level in the Tg2 line with 19 copies did not even reach the *Camk2a* endogenous levels. As such, the transgene copy number did not correlate with the transgene expression levels in the two independent lines (Supplementary Figures 6A,B).

## Discussion

Based on previous work in chicken it has been proposed that activation of the endogenous CaMKII activity controls the onset of the prehypertrophic and hypertrophic chondrogenic program. In proliferating chondrocytes, the phosphorylated form of CaMKII is not detectable and possibly constantly dephosphorylated under the influence of PTHrP signaling (Li et al., 2011). As a consequence, the phosphorylated form of CaMKII is limited to the prehypertrophic and hypertrophic chondrocytes in chicken and mouse (Li et al., 2011). In the chicken, ectopic expression of an activated form of CaMKII, was able to override the endogenous CaMKII activity or lack thereof in proliferating chondrocytes and led to premature and ectopic activation of the prehypertrophic/hypertrophic program in cells outside of the normal maturation zones (Taschner et al., 2008; Li and Dudley, 2009; Li et al., 2011). In mouse long bone development, the phenotypic effects of CaMKII activation in proliferating, type II collagen-expressing chondrocytes were very mild and *in vivo*, effects could only be detected developmentally around the onset of chondrocyte hypertrophy at E12.5–E13.5. Here, our data suggest that in agreement with previous findings in chicken, DaCaMKII in mouse activated the prehypertrophic/hypertrophic program prematurely at early stages of endochondral ossification, while down-regulation of endogenous CaMKII activity interfered with the onset of the prehypertrophic and hypertrophic program. Interestingly, the phenotypic changes caused by CaMKII activity modulation at the onset of chondrocyte maturation did not accumulate over time despite the fact that the transgene was continuously expressed in type II collagen-producing chondrocytes (Supplementary Figure 5). Yet, in contrast to the overexpression experiments in chicken, no premature maturation of chondrocytes outside their normal maturation zones or shortening of the limbs was observed. Hence, the phenotypic consequences of ectopic activation of CaMKII are quite distinct between chicken and mouse (Taschner et al., 2008; Li and Dudley, 2009; Li et al., 2011). Possible explanations for the phenotypic discrepancies between chicken and mouse could be that on the one hand in the chicken not only chondrocytes also the soft tissue is infected and that this could contribute to the phenotype. On the other hand, it is likely that the retroviral driven expression levels of the transgenes were much higher in chicken. For retroviral driven transgenes over 100-fold expression levels have been reported (Geetha-Loganathan et al., 2014; Nimmagadda et al., 2015). In the mouse, we did not detect phenotypic differences between the independent transgenic lines despite the fact that they varied in copy numbers (Figure 3A), which can be explained by the fact that transgene expression levels were similarly low in both cases (about one-fold of the Camk2a endogenous levels; Supplementary Figure 6). Hence, the lack of a major phenotype in the transgenic mice may be associated with the relatively low transgene expression levels compared to the expression levels that have been reached by retroviral expression in the chicken. Although, of course, the actual levels of retroviral-driven DaCaMKII transgene expression were not determined in the chicken experiments performed previously (Taschner et al., 2008; Li and Dudley, 2009; Li et al., 2011). A possible explanation for the observed transient phenotype at the early stages of skeletal development maybe that the onset of chondrocyte maturation may present a window of opportunity for the transgenes to excert a mild effect accelerating, respectively delaying chondrocyte maturation. And that this effect is later on compensated as the chondrocyte maturation program comes under the transcriptional control of many regulatory factors and feed back mechanisms as development progresses (Hartmann, 2009; Kozhemyakina et al., 2015).

At the molecular level, we observed *in vitro* that CaMKII robustly affects the transcriptional activity of the transcription factor Mef2c and to a lesser extent also Runx2 transcriptional activity. Mef2c and Runx2 start to be expressed as the chondrocytes undergo hypertrophy (Arnold et al., 2007). Mef2c and Runx2 activity are both negatively regulated by the class II histone deacetylase HDAC4 (Vega et al., 2004; Kozhemyakina et al., 2009; Correa et al., 2010). In vascular smooth muscle cells, CaMKIIdelta2 regulates Mef2 transcriptional activity through HDAC4/5 (Ginnan et al., 2012). Yet, our results indicate that activated CaMKII increases Mef2c activity by an HDAC4-independent mechanism. As the activated form of CaMKII physically interacts with Mef2c, the underlying mechanism may involve phosphorylation of Mef2c protein by CaMKII. In different cell types other kinases, such as p38 MAPK and ERK5, have also been shown to phosphorylate Mef2c enhancing its transcriptional activity (Han et al., 1997; Kato et al., 1997). Yet, there is the obvious discrepancy between the *in vitro* results where a robust stimulatory effect on Mef2c transcriptional activity was observed and the subtle *in vivo* effects. For the *in vitro* luciferase experiments an expression vector was used that drives DaCaMKII expression under the control of the CMV promoter, which drives high levels of expression in mammalian cells. The high transgene expression levels *in vitro* (Supplementary Figure 1) may be an explaination for the strong *in vitro* effect on Mef2c activity and in contrast to the only mild effect *in vivo* where transgene expression levels were at least a 100-fold lower. Furthermore, Li and colleagues proposed that the endogenous CaMKII activity is opposed by an inhibitory gradient, which is under the control of PTHrP signaling (Li et al., 2011). PTHrP signaling via cAMP and protein kinase A negatively regulates chondrocyte hypertrophy through HDAC4 mediated inhibition of Mef2c activity (Kozhemyakina et al., 2009). Yet, even *in vitro*, DaCaMKII signaling was not able to overcome or to partially revert the effect of HDAC4 on Mef2c activity (Figure 2C). The PTHrP expression itself is under the regulatory control of Ihh-signaling (Vortkamp et al., 1996; St-Jacques et al., 1999). As such the slightly increased Ihh expression observed in the E12.5 DaCaMKII transgenic limbs may upregulate PTHrP signaling and counteract a possibly direct, positive effect of DaCaMKII on the Mef2c activity via HDAC4. Hence, one possible scenario may be that increased levels of PTHrP signaling override the activation of Mef2c by DaCaMKII at later stages of development.

## Author contributions

CA: Data collection, assembly, analyses, interpretation, and manuscript writing. CF: Performed the co-immunoprecipitation studies. AH: Performed qPCR studies determining transgene copy numbers and expression levels, and proof-read the manuscript. LW: Performed histological analysis on E18.5 material. CH: Conception and design, data interpretation, and manuscript writing.

## Funding

Part of the work was conducted at the Institute of Molecular Pathology in Vienna which is funded by Boehringer Ingelheim. AH was supported by a grant from the Deutsche Forschungsgemeinsaft [HA 4767/2-1] and LIW is supported by a grant from the BMBF [Overload-PrevOP, SP07]. The funding sources had no role in study design, data collection and analysis, decision to publish, or preparation of the manuscript.

### Conflict of interest statement

The authors declare that the research was conducted in the absence of any commercial or financial relationships that could be construed as a potential conflict of interest.

## Abbreviations

CaMKII: Calcium/calmodulin-dependent kinase II
Gfp: Green fluorescent protein
ISH: in situ hybridizations
tg: transgenic
E: embryonic day.
