# Supplementary material for: CaMKII Signaling Stimulates Mef2c Activity In Vitro but Only Minimally Affects Murine Long Bone Development in vivo
Source: Front Cell Dev Biol. 2017 Mar 16;5:20. doi: 10.3389/fcell.2017.00020 (PMC5352711; doi:10.3389/fcell.2017.00020)
Supplement: Supplementary file 1 [file DataSheet1.pdf]

Supplementary Material

CaMKII Signaling Stimulates Mef2c Activity In Vitro But Only Minimally Affects Murine Long Bone Development In Vivo

Chandra Sekhar Amara[1], Christine Fabritius[1], Astrid Houben[1], Lena Ingeborg Wolff[1], Christine Hartmann[1]*

[1] Institute of Experimental Musculoskeletal Medicine, Dept. Bone and Skeletal Research, Medical Faculty of the University of Münster (WWU), Münster, Germany

*** Correspondence:** Christine Hartmann: christine.hartmann@ukmuenster.de

1 Supplementary Figures and Tables

1.1 Supplementary Figures

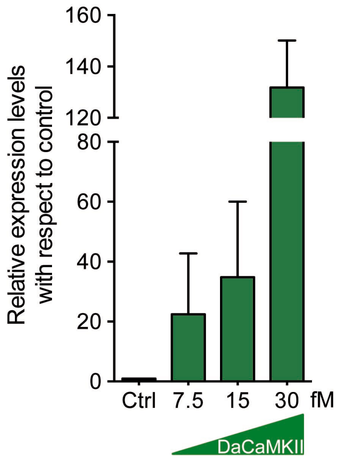

Supplementary Figure 1. Bar graph showing the qPCR results for the *Camk2a* expression levels in control transfected chondrocytes and chondrocytes transfected with increasing amounts of DaCaMKII used for the luciferase experiments shown in Figure 1C-F. Error bars ±SEM, n=2.

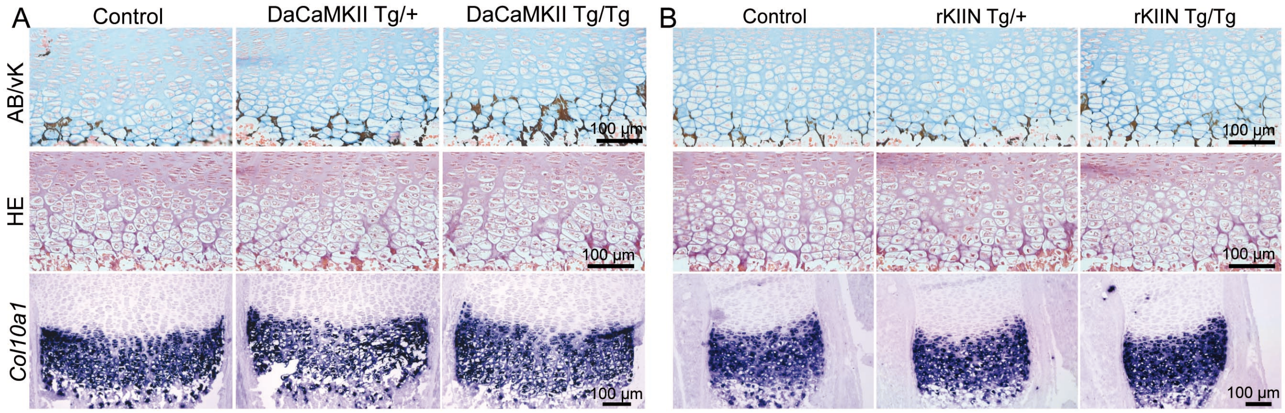

Supplementary Figure 2. **(A)** Representative images of sections through the proximal growth plate of E18.5 humeri of control (non-transgenic) and DaCaMKII transgenic (heterozygous and homozygous) littermates stained with alcian blue / von Kossa (AB/vK), hematoxylin / eosin (HE), and ISH with a *Col10a1* riboprobe. **(B)** Representative images of sections through the proximal growth plate of E18.5 humeri of control (non-transgenic) and rKIIN transgenic (heterozygous and homozygous) littermates stained with alcian blue / von Kossa (AB/vK), hematoxylin / eosin (HE), and ISH with a *Col10a1* riboprobe.

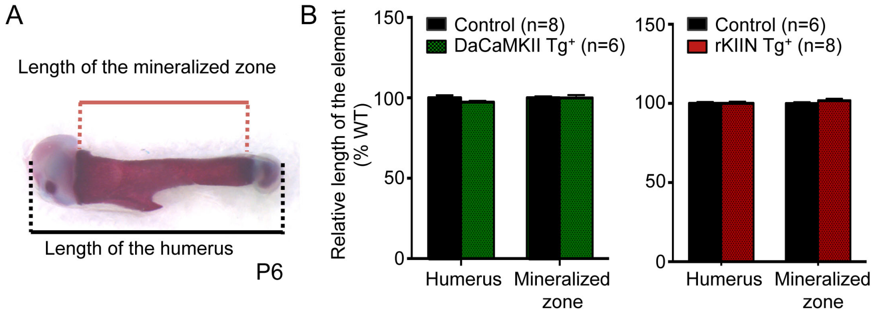

Supplementary Figure 3. (A) Exemplary alcian blue / alizarin red stained humerus from a 6-day old pup (P6) used to determine the following parameters: overall length of the skeletal element (indicated by the black line) and the length of the maturation zone (indicated by red line). The measurements were performed using Metamorph software with defined pixel settings. **(B)** Relative ratios between the lengths of skeletal elements and lengths of maturation zones of humeri between DaCaMKII-, rKIIN-tg mice, and their non-transgenic control littermates. *n* refers to the number of independent biological samples. Error bars indicate ±SEM.

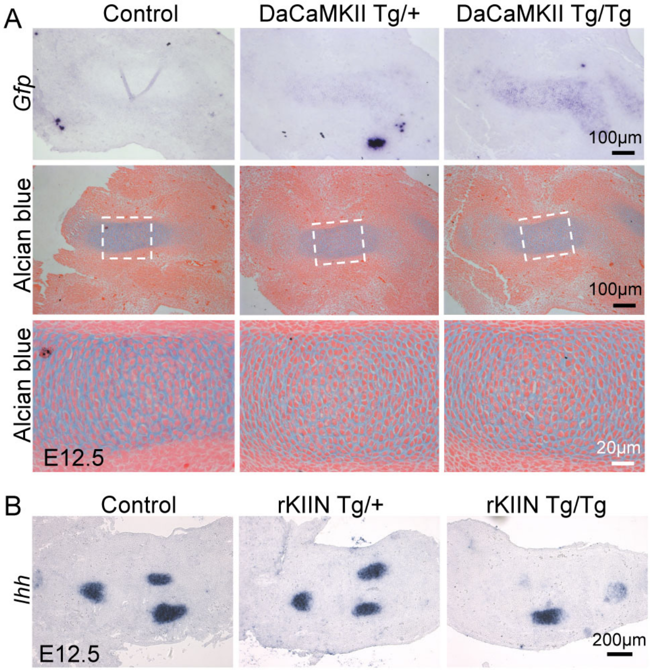

Supplementary Figure 4. (A) Representative images of sections through E12.5 forelimbs of respective control (non-transgenic) and DaCaMKII-tg littermates hybridized with a *Gfp* anti-sense riboprobe to distinguish control limbs from hetero- and homozygous transgenic animals. Shown below, alternating sections stained with alcian blue to assay histological changes. Bottom panel: magnifications of the boxed areas in the images of the panel above. **(B)** *Ihh* ISH on sections through E12.5 forelimbs of control (non-transgenic) and rKIIN-tg littermates showing no changes in the signal intensity or the domain size comparing transgenic to non-transgenic littermate control limbs.

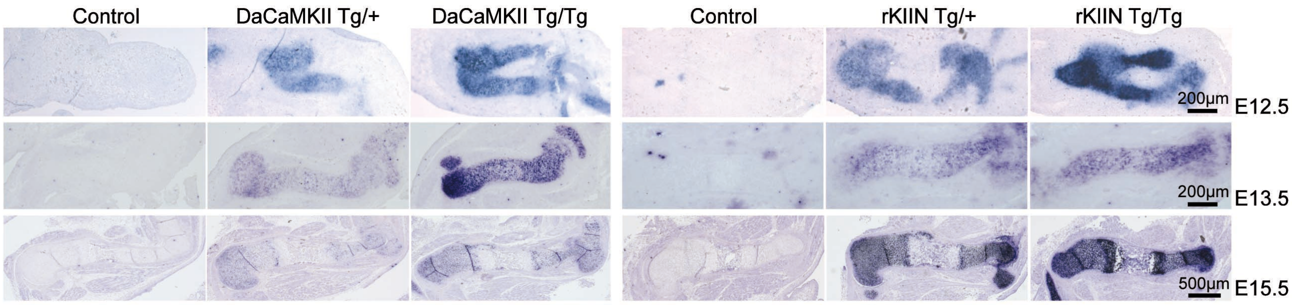

Supplementary Figure 5. ISH with a *Gfp* riboprobe on sections through control (+/+), heterozygous (Tg/+), and homozygous (Tg/Tg) transgenic E12.5, E13.5, and E15.5 limbs of control, DaCaMKII and rKIIN transgenic mice revealed transgene expression at all developmental stages corresponding to the *Col2a1* expression domain. Note: no transgene expression is detected in the respective control, non-transgenic littermates.

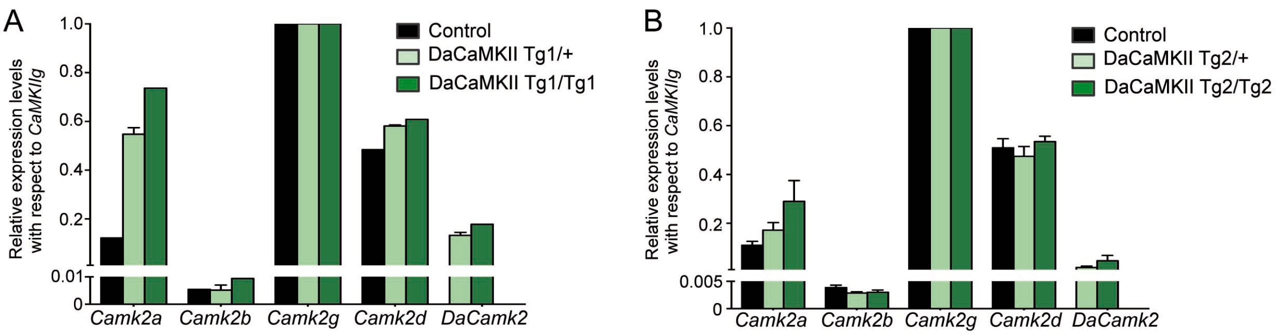

Supplementary Figure 6. (A) Bar graph showing the relative expression levels of the four *Camk2* isoforms, α, β, γ, δ, and of the *DaCamk2-eGFP* transgene in the skeletal elements of littermate controls and E12.5 embryos of the transgenic line DaCaMKII-Tg #1 (Tg1). (**B)** Bar graph showing the relative expression levels of the four *Camk2* isoforms, α, β, γ, δ, and of the *DaCamk2-eGFP* transgene in the skeletal elements of littermate controls and E12.5 embryos of the transgenic line DaCaMKII-Tg #2 (Tg2). Note: The expression of the endogenous *Camk2a* isoform increased in the skeletal element of the transgenic line #1. Error bars indicate ±SEM, n=2-3.

1.2. Supplementary Tables

Mouse line	Primer sequences
Col2a1-DaCaMKII-eGFP	`5′-GAGGACGTCGACTGCCTGAAG-3′`<br>`5′-GGTCAGCTTGCCGTAGGT-3′`
Col2a1-eGFP-RKIIN	`5′-GGTCAGCTTGCCGTAGGT-3′`<br>`5′-AACCATGTTCATGCCT-3′`

Supplementary Table 1: Primer sequences for genotyping of transgenic mice.

Gene	Primer sequences	Product size (bp)
DaCaMKII-eGFP	`5′-TGCCCCATGGTTCTGGTTCT-3′`<br>`5′-TAGCGGCTGAAGCACTGCAC-3′`	250
eGFP-RKIIN	`5′-TCCTGCTGGAGTTCGTGACC-3′`<br>`5′-ACGGGCAGAGGCTAGCTGC-3′`	152
Actb	`5′-TGGGAATGGGTCAGAAGGACT-3′`<br>`5′-GGGTCATCTTTTCACGGTTGGC-3′`	228

Supplementary Table 2: Primer sequences used for determination of transgene copy number by qPCR.

Gene	Primer sequences	Product size (bp)
Ihh	5′-CGTGCATTGCTCTGTCAAGT-3′<br>5′-GTGGGCTGCTGGTTCTGTAT-3′	313
Col10a1	5′-TGAACGGTACCAAACGCCCACAG-3′<br>5′-TTCCCCTTTCCGCCCATTCACAC-3′	505
Axin2	5′-AAGCCTGGCTCCAGAAGATCACAA-3′<br>5′-TTTGAGCCTTCAGCATCCTCCTGT-3′	134
Lef1	5′-CCACACGGACAGTGACCTA-3′<br>5′-GCCCAGGATCTGGTTGATAG-3′	184
Mef2c	5′-AGATCTGACATCCGGTGCAG-3′<br>5′-TCTTGTTCAGGTTACCAGGT-3′	98
Runx2	5′-CCCAGGCGTATTTCAGATGA-3′<br>5′-TAGTGCATTCGTGGGTTGGA-3′	176
Camk2a	5′-TGGAGACTTTGAGTCCTACAC-3′<br>5′-TTCCGGGACCACAGGTTTTC-3′	129
Camk2b	5′-GTTTCACCGACGAGTACCAG-3′<br>5′-GCGTACAATGTTGGAATGCTTC-3′	200
Camk2g	5′-GTTTGACCTTGTTACCGGAGG-3′<br>5′-CACCCTTGCATTTACTCGCC-3′	185
Camk2d	5′-GATCAAGGCCGGAGCTTACG-3′<br>5′-AGAGGCTGTGATACGTTTGGC-3′	118
DaCamk2-eGFP	5′-TGCCCCATGGTTCTGGTTCT-3′<br>5′-TAGCGGCTGAAGCACTGCAC-3′	250
Gapdh	5′-AGGTCGGTGTGAACGGATTTGG-3′<br>5′-TTGATGTTAGTGGGGTCTCGCTCC-3′	244
Actb	5′-TTGCTGACAGGATGCAGAAGGAGA-3′<br>5′-ACTCCTGCTTGCTGATCCACATCT-3′	159

Supplementary Table 3: Sequences of primer pairs used for qPCR. All primer pairs with the exception of the primers used to detect expression of the *DaCamk2-eGFP* transgene are spanning exon-intron boundaries.
